# The influence of modality on input, visuo-motor coordination, and execution in the advanced pianist's sight-reading processes

**DOI:** 10.3389/fpsyg.2022.933106

**Published:** 2022-07-26

**Authors:** Jing Qi, Mayumi Adachi

**Affiliations:** Department of Psychology, Hokkaido University, Sapporo, Japan

**Keywords:** sight-reading, modality, fixation, eye-hand span, errors

## Abstract

In this study, we explored how the modality (major and minor) would affect the input (i. e., fixation), visuo-motor coordination (i.e., eye-hand span and time of performance), and execution (i.e., errors) in the advanced pianist's sight-reading processes, as well as relations among these three phases. Thirty-two advanced pianists with 5–54 years of piano training participated in the study. All participants sight-read three two-voice pieces in either major (*n* = 16) or minor (*n* = 16) mode while their eye movements were measured by an eye-tracking device (30 fps). All pieces were 20-measure long written in 4/4 m, adapted from unfamiliar Baroque pieces. Results showed that sight-readers fixated more frequently and tended to spend more time performing in a minor score than in a major score. This implies that modality of a score affects an efficiency of input and visuo-motor coordination in the advanced pianist's sight-reading. Spearman's correlation coefficients showed that errors were correlated positively with the number of fixations and the duration of performance. These results add more evidence to the notion that efficiencies in input and visuo-motor coordination are related to the accuracy in execution.

## Introduction

Music sight-reading is an indispensable skill for professional musicians, often required to perform an unfamiliar score without any practice. This skill involves complex processes related to many different factors such as a performer's cognitive ability, motor skills, memory, musical ability, and music training (Wolf, 1976; Chang, 1993; Lehmann and Kopiez, 2009; Zhukov, 2014b; Rosemann et al., 2016). Particularly unique in music sight-reading is its involvement of an information conversion process from visual to motor domains (Sloboda, 1983). While performers process music information in a score through vision, their somatosensory cortex executes muscle movements to generate the appropriate sound (Inhoff and Gordon, 1998; Kaketa, 1998). Many researchers have worked tirelessly to be able to elucidate the whole process of sight-reading. This is a complex process that involves at least two types of skills: reading skills and mechanical skills (Wolf, 1976). It means that performers must encode the musical information presented on the score into the brain (i.e., reading skill), then they need to place the fingers in the right position at the right time and execute it (i.e., mechanical skill). The process of sight-reading also involves auditory feedback (Banton, 1995), because in the end the performer needs to confirm by listening to whether the encoded musical information is consistent with the output. Therefore, it seems that the cognitive processing of sight-reading can be divided into three phases: visual input, visual-to-motor sensory conversion, and execution. In the following, we will describe how each of these three processing phases of sight-reading works and how to evaluate their efficiency.

The first phase called *input*. Sight-reading and text-reading possess similar cognitive processing (Sloboda, 1977; Chang, 1993). In fact, when reading text, our eyes do not operate in a single sustained photographic action like a digital camera, but by projecting the scene reflected in the pupil onto the retina through jumping, discrete eye movements. This is similar to movements such as turning a flashlight on and off at short intervals in the dark (Lehmann and Kopiez, 2009, p. 344). This discrete eye movement is called saccade, and the average length of each saccade is about 5–9 letters in text reading. A temporal pause in eye movement between two discrete saccades is called *fixation*, through which readers encode text information. In music, sight-readers briefly suspend eye gazes at particular locations of a sore to encode information on the score. There is a large difference in eye movements of good sight-readers from those of poor sight-readers. For example, the mean duration of fixation is ≈350–400 ms (Rayner and Pollatsek, 1997). A good sight-reader uses less fixation and shorter duration of fixation in sight-reading a music piece (Goolsby, 1994; Waters et al., 1997; Drai-Zerbib et al., 2012). These and further experimental studies (Furneaux and Land, 1999; Lehmann and McArthur, 2002; Penttinen and Huovinen, 2009) support the classical notion, derived from interviews and observations of professional pianists (Wolf, 1976), that good sight-readers input necessary information in a greater chunk than poor sight-readers and increase the efficiency of visual information processing. On the other hand, the sight-reader not only looks at the score when sight-reading, but also looks down at the keyboard to check the position of the fingers (Banton, 1995). For example, when the sight-reader is unsure of a note to be played or a note that has already been played, he or she will habitually look down at the keyboard. Such an action tends to affect the sight-reader's fixation, making it necessary to pause to look at the musical information, resulting in less efficient input. The efficiency (or inefficiency) of input can also be measured by the duration of performance: A longer performance means a slower tempo that can result in a longer duration and a greater number of fixation (Chang, 1993; Furneaux and Land, 1999; Lim, 2018), which can imply a low efficiency in visual processing.

The duration of performance can also indicate an efficiency of the second phase of sight-reading process, *visuo-motor coordination*. In this phase, sight-readers transform visual information from a score to motor functions so that their hands (or vocal cords) can work appropriately (Rayner, 1998; Adachi et al., 2012). A shorter duration of performance can indicate faster processing of visuo-motor coordination, implying a temporal efficiency in sight-reading. Another measure for the efficiency of visuo-motor coordination is *eye-hand span* (or *EHS*), the distance between a note being played (i.e., hand position) and that being looked ahead (i.e., eye position) (Sloboda, 1974; Rayner and Pollatsek, 1997; Truitt et al., 1997; Furneaux and Land, 1999; Lehmann and McArthur, 2002; Adachi et al., 2012; Rosemann et al., 2016). It has been acknowledged that good sight-readers look further ahead of the notes that are being played (e.g., Sloboda, 1974, 1977; Goolsby, 1994). EHS can be measured by either *time index* (i.e., the duration between a note being fixated and that note being played) or *note index* (i.e., the number of notes looked ahead while playing a particular note). With time index, the average of EHS has been reported ≈0.70–1.48 s in music sight-reading (Furneaux and Land, 1999; Wurtz et al., 2009; Rosemann et al., 2016; Lim, 2018). With note index, EHS can vary depending on the type of music and the skill of sight-reader (Chang, 1993; Furneaux and Land, 1999; Gilman and Underwood, 2003). For example, according to a study by Weaver (1943), EHS for a four-voice chorale-like music has been reported as 1.5 notes, that for a homophonic music (i.e., a melody with its accompaniment) as 1.9 notes, and that for a two-voice contrapuntal music as 3.1 notes. EHS of good sight-readers can be as long as 6.8 notes while that of poor sight-readers can be only 3.8 notes (Sloboda, 1974).

While sight-readers coordinate between visual and motor domains, they also make actions and generate the intended sound (e.g., move their physical parts on an instrument or use their vocal cords and other related muscles). This is the third phase of sight-reading process, *execution*. An accuracy of execution has been measured by the number of errors, which evaluates a degree of accuracy while executing information transformed from visual to motor domain (Sorel and Diamond, 1968; McPherson, 1994; Gilman and Underwood, 2003; Highben and Palmer, 2004; Gudmundsdottir, 2010; Adachi et al., 2012; Zhukov et al., 2016). The number of errors is often used to differentiate sight-readers between good and poor (Drake and Palmer, 2000; Gilman and Underwood, 2003; Besson et al., 2007). Good sight-readers tend to make fewer errors in execution and have a higher accuracy rate than poor sight-readers. This seems to be because skilled sight-readers are more efficient, they can perform correctly without interruptions and tend to make fewer errors (Drake and Palmer, 2000; Herrero and Carriedo, 2019). Moreover, the number of errors depends on the style of music (Chang, 1993): Sight-readers make more errors in contemporary (13 %) than contrapuntal Baroque music (6%). Moreover, *stuttering*—trying to correct mistakes by playing more than once—can be an index for flow of execution that may relate to one's confidence in sight-reading. For example, the beginners make more stuttering than the advanced sight-readers; moreover, the advanced sight-readers stutter more often in sight-reading a score with complex meter (Adachi et al., 2012).

Thus, previous research on sight-reading has provided us with various measures that can evaluate the accuracy and the efficiency of input and visuo-motor coordination as well as the accuracy and the flow of execution. Any of these individual measures could highlight what differentiates between good and poor sight-readers; however, it is not fully understood how different abilities across the three phases relate to each other. In fact, such information is scarcely explored except in a few studies. For example, EHS with note index is correlated negatively with the number of errors (Sloboda, 1974; Gilman and Underwood, 2003; Rosemann et al., 2016; Cara, 2018), which implies that the efficiency of visuo-motor coordination may be responsible for the accuracy of execution. It may be vague just to show that the number of errors or stuttering decreases as the level of expertise (or musical training) increases (e.g., Penttinen and Huovinen, 2009; Adachi et al., 2012; Zhukov, 2014a,b; Zhukov et al., 2016), but this information should become noteworthy if one can demonstrate the origins of errors or stuttering to be different between expertise (or training) levels. For example, the number of the beginner's stuttering is correlated negatively with the proportion of fixation (implying its origin to be an inefficiency in input), whereas that of the advanced pianist's is correlated positively with the numbers of pitch and rhythm errors (implying its origin to be a failure in execution) (Adachi et al., 2012). Thorough examinations of relations between measures across input, visuo-motor coordination, and execution will fill in the void in our knowledge of mechanisms of music sight-reading. Moreover, we found that most past studies on sight-reading were performed under restricted rhythmic conditions. In reality, however, sight-reading is not usually performed under restricted rhythms. This finding made us realize that the efficiency of information processing is likely to be affected when sight-reading is performed under rhythm-constrained conditions. Therefore, allowing the sight-reader to perform sight-reading at a natural, unrestricted tempo can maximize the recovery of the sight-reading information processes.

In addition, in the same composition, the minor mode passage is played more slowly than the major mode passage (Post and Huron, 2009). This is probably because the performers have to play at a slower tempo due to less efficient processing of information when playing a minor mode passage. However, there does not seem to be a difference in the proportion of errors between major and minor modes (Lewandowska and Schmuckler, 2020). We suggest that this may be because the reduction in performance speed relieves the cognitive load on the sight-reader and improves the accuracy of the performance. Our review of 47 music sight-reading studies from 1968 to 2018 have revealed that 45 used scores in major while only 14 used those in minor (Qi and Adachi, 2022). Of those, 12 used both major and minor, but none compared multiple dependent variables between these two modes such that their results could capture an overall picture of sight-reading process in tonal music.

The purpose of the present study was two-folds: (1) to investigate effects of modality (major and minor) in three phases of sight-reading process (i.e., input, visuo-motor coordination, and execution) observed on the piano, and (2) to identify directional relations between variables obtained across these phases. In particular, we focused on sight-reading by the advanced pianists (i.e., the population most often studied in the literature), using two-voice contrapuntal music that tends to elicit more efficient visuo-motor coordination than other styles of music (Weaver, 1943; Chang, 1993; Rayner and Pollatsek, 1997). Moreover, we incorporated the complexity of intervallic relations of notes in each voice by preparing for comparable scores between major and minor materials, since the predictability of upcoming intervals would influence the efficiencies of input and visuo-motor coordination as well as the accuracy of execution (Chang, 1993; Ronkainen and Kuusi, 2009).

We predicted that, first, sight-reading a major score would lead to more efficiencies in the process of input and visuo-motor coordination than a minor score, since the latter generally contains more accidentals than the former, but the number of errors in execution would be equivalent between two modes (Post and Huron, 2009). Second, sight-reading a more complex score would result in lower efficiencies in all three phases as well as less accuracy and flow in execution (Chang, 1993; Ronkainen and Kuusi, 2009). Finally, an efficiency in visuo-motor coordination would be correlated negatively with the errors (i.e., higher accuracy and flow) in execution (Sloboda, 1974; Gilman and Underwood, 2003; Rosemann et al., 2016; Cara, 2018). Our analyses of other relations across phases were exploratory without any specific prediction. Together, we hoped not only to verify previous findings but also to touch upon unclear issues that had rarely been discussed in music sight-reading research.

## Method

### Participants

To gather more participants for the experiment, a request was sent to a piano professor at the School of Music to recommend proficient performers of the piano. Specifically, they are undergraduate piano major students and pianists who have continued their musical activities after graduation, as well as undergraduate students who are not majoring in piano (i.e., majoring in composition, music education, etc.), who have received systematic piano instruction, participated in piano competitions, and have been judged to have professional performance ability. Finally, 32 advanced pianists with healthy (or corrected) vision participated in this study. The sample consisted of 24 women and eight men, ranging in 18–58 (*M* = 21.72, *SD* = 7.60) years of age, with 5–54 (*M* = 14.11, *SD* = 8.02) years of formal piano training. Prior to the experiment, each participant signed a consent form explaining its purpose, procedure, and possible risks as well as informing a permission to withdraw anytime as wished. Participants received 3,000 JPY or small gifts upon completion as incentive.

### Scores

Two scores (consisting of 2-octave scale, arpeggio, and cadence) were prepared for a warm-up exercise in E major (for those who sight-read major scores) and f minor (for those who sight-read minor scores), which uses four sharps and four flats in their key signatures, respectively. For a practice trial, two unfamiliar scores—G major and c minor—were created, based on unknown fugues by George Frederick Handel (1685–1759). For trials, three unfamiliar scores with different complexities of intervallic relations between notes were created for each modality (three scores for major condition and the other three scores for minor condition), based on unknown pieces by Johann Ernst Bach (1722–1777) and George Frederick Handel. All scores were polyphonic (i.e., two voices) in 4/4 m consisting of 20 measures arranged in four rows in one page, generated on Finale 2014 (version 2014d.v5545, makemusic).

To control the difficulty of scores between major and minor conditions, we calculated the complexity of intervallic progressions per score by applying the concept of melodic expectancy. Music expectancy was first introduced by a music theorist Meyer (1956) as a listener's anticipatory state of mind toward upcoming musical events while music is being played, and its bottom-up mechanism, especially, in melody has been investigated psychologically (Carlsen et al., 1970, 1994; Carlsen, 1981, 1982; Adachi, 1995) and developed into different models (e.g., Krumhansl, 1995; Schellenberg, 1997). We used one of principles in two-factor model by Schellenberg (1997): pitch proximity (PP). The principal PP represents that listener would anticipate (or expect) smaller intervals more than large intervals in upcoming musical events (Schellenberg, 1997), originally deriving from Meyer's theory of melodic expectancy based on Gestalt principle (Meyer, 1956). Musicians' strong expectancies for particular musical events can make them ignore misprints on a score, allowing them to sight-read while self-correcting notes in the way the composer would have intended (Sloboda, 1977, 1985). This suggests that scores consisting of intervals more likely to be anticipated (e.g., smaller intervals) can reduce cognitive load of bottom-up processes, resulting in easier sight-reading than otherwise. Based on this speculation, we developed an index called the intervallic complexity, representing mean expected value of an upcoming interval per score. The following is how to calculate.

First, we calculated the probability of occurrence of interval “E” —P(E)— in the target score as follows:


$$\begin{array}{l} P\left(E\right)=\;\frac{Frequency\;of\;”E”}{Total\;Number\;of\;Intervals} \end{array}$$

Second, we calculated *entropy*, i.e., a value indicating how uncertain it is for a particular event (or, in this case, a particular interval) to occur (Meyer, 1957; Carlsen et al., 1994). The greater I(E) is, the more uncertain (thus less expected) the upcoming interval would be. The entropy for interval “E” —I(E)—can be defined as:


$$I(E)\;=log_{2}\;(\frac{1}{P(E)})=\;-P(E)$$

Third, we calculated uncertain value of interval “E” —UV(E)—as an upcoming note while applying I(E) and an index of pitch proximity—PP_E_–expressed as the number of semitones between two adjacent notes. Here, 1 is added to PP_E_ to avoid UV to be 0 when PP_E_ is 0 (i.e., when the same pitch is repeated in a score).


$$\begin{array}{l} UV\left(E\right)=\left(PP_{E}+1\right)\times I\left(E\right) \end{array}$$

Finally, mean of UV for a particular score was calculated as follows:


$$\begin{array}{l} Mean\;of\;UV=\;\frac{Total\;of\;UV}{Total\;Number\;of\;Intervals\;used\;in\;the\;target\;score} \end{array}$$

A larger mean of UV indicates a score to be more complex such that sight-readers cannot anticipate upcoming intervals as easily as those with a smaller mean of UV.

The intervallic complexity (i.e., mean of UV) of each score is shown in Table 1. Bootstrap paired *t*-tests revealed that three levels of intervallic complexity were significantly different from each other: *t*_*obt*_(1) = 2.08, *p* = 0.002, *d* = 2.91 (high vs. medium); *t*_*obt*_ (1) = 5.45, *p* = 0.002, *d* = 2.47 (medium vs. low); *t*_*obt*_ (1) = 4.10, *p* = 0.002, *d* = 5.24 (high vs. low). Moreover, another bootstrap paired *t*-test revealed no significant difference in the intervallic complexity between major and minor conditions, *t*_*obt*_ (2) = 0.05, *p* > 0.05, *d* = 0.02.

**Table 1:** The intervallic complexity of each score identified with the left letter as the level of complexity and the right letter as modality.

	**Intervallic Complexity**			
**Modality**	**High**	**Medium**	**Low**	***M (SD)***
Major	HM (B^b^): 3.04	MM (F): 2.48	LM (G): 1.77	2.43 (0.64)
Minor	Hm (a): 3.65	Mm (g): 2.04	Lm (g): 1.55	2.42 (1.10)

*Each cell consists of score ID with the tonic in parenthesis, followed by the value of intervallic complexity*.

*^b^Stands for flat*.

### Apparatus

Each participant performed sight-reading tasks on an electronic piano (MP-300, Roland) while wearing a baseball-cap type eye-tracking device (EMR-9, Nac) to record eye movements. The sound from the electric piano was recorded directly to the controller of EMR-9 so that its timing was synchronized with the recorded eye movements. The experiment was controlled by a program created on PsyScope X (Cohen et al., 1993) through a laptop computer (MacBook Air, OS10.13, Apple). Verbal instructions and scores were presented on a 19-inch display (L1919C-BFS, LG), positioned at the score stand of the electric piano. An additional digital video camera (HF-R62, Canon) captured each participant's postures and behaviors throughout the experiment.

The recorded eye movements were imported to a laptop computer (MacBook Pro, Windows 7 Ultimate Service Pack 1) where the number of fixations was identified on a software (EMR-dFactory, version 2.71a, Nac). To identify eye-hand span with note index (i.e., N-EHS), the recorded sound was first converted to wave form using Final Cut Pro (version 10.4.1, Apple), and then the wave form of the note being played at the time of each fixation (indicated by dFactory) was identified manually by checking eye movements frame by frame, using iMac (OS High Sierra 10.13.3, Apple) with a 27-inch Retina display (5,120 × 2,880).

### Procedure

The experiment was executed in a quiet room. In the beginning, each participant played a well-practiced piece of their choice as a warm-up. Then, an eye tracker was attached and calibrated to each eye. To get used to playing a score while maintaining their head position, participants first played a simple score consisting of ascending and descending scales followed by arpeggios and cadence, and then sight-read either a major or a minor score as a practice trial. Those who were assigned for major condition (*n* = 16) played scores in a major key both for scales and practice trials while others (*n* = 16) played them in a minor key.

For experimental trials, each participant sight-read 3 two-voice scores, written in an assigned mode, presented randomly. Each trial began with presentation of a score followed by 1-min preview during which any preparation except pressing piano keys was allowed (e.g., singing/humming a melody, moving fingers on the lap, tapping beat). A chime sound was presented as a sign of the end of preview. Upon completion of each sight-reading, the participants responded to two questions by 9-point scale (1 as “not at all” to 9 as “completely”): (1) how much they could prepare during 1-min preview and (2) how difficult it was to sight-read. In addition, the participants responded whether they had played the given score before. Each participant's personal information including past musical experiences and training activities during 1 year prior to the experiment was obtained through questionnaire. The entire experiment including a warm-up lasted ≈60 min.

### Analysis

In the present study, we analyzed fixations, eye-hand span, duration of sight-reading performance, and errors in performance. Two rows in the middle of each score (i.e., measures 6–15) were the target of analysis due to possible deviations in recorded eye movements near top and bottom edges of the display. Each dependent variable was measured as follows.

#### Fixation

**Figure 1:**
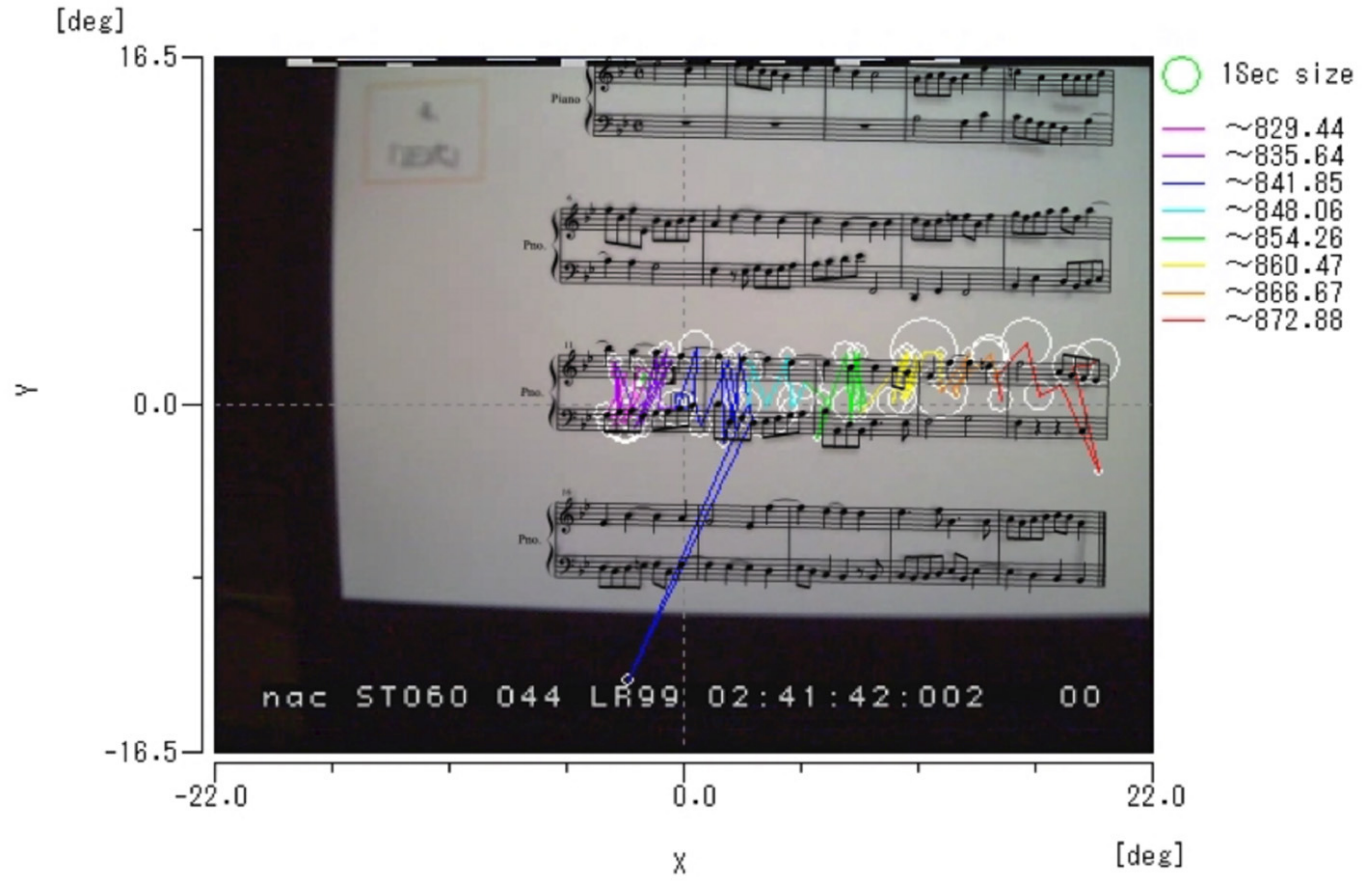
An example of visualization of eye tracking while one participant was playing the third row of score HM. The size of white circle represents the duration of each fixation with the size of green circle on the right column as 1 s (i.e., 1,000 ms). Lines with different colors show traces of eye tracking in time lines shown on the right column.

For the efficiency of input, we analyzed fixation as a measure for the participant's eye movements. Either left or right eye movement (recorded more consistently than the other side) was used in measurements. A fixation was defined as the eye movement staying at the same location equal to or longer than 100.00 ms (see Figure 1). Three dependent variables were obtained: (1) the number of fixations, (2) the mean number of frames per fixation, and (3) the proportion of fixations. The eye movements were identified frame by frame with 1 frame equals to 33.33ms.

#### Eye-hand span and the duration of performance

**Figure 2:**
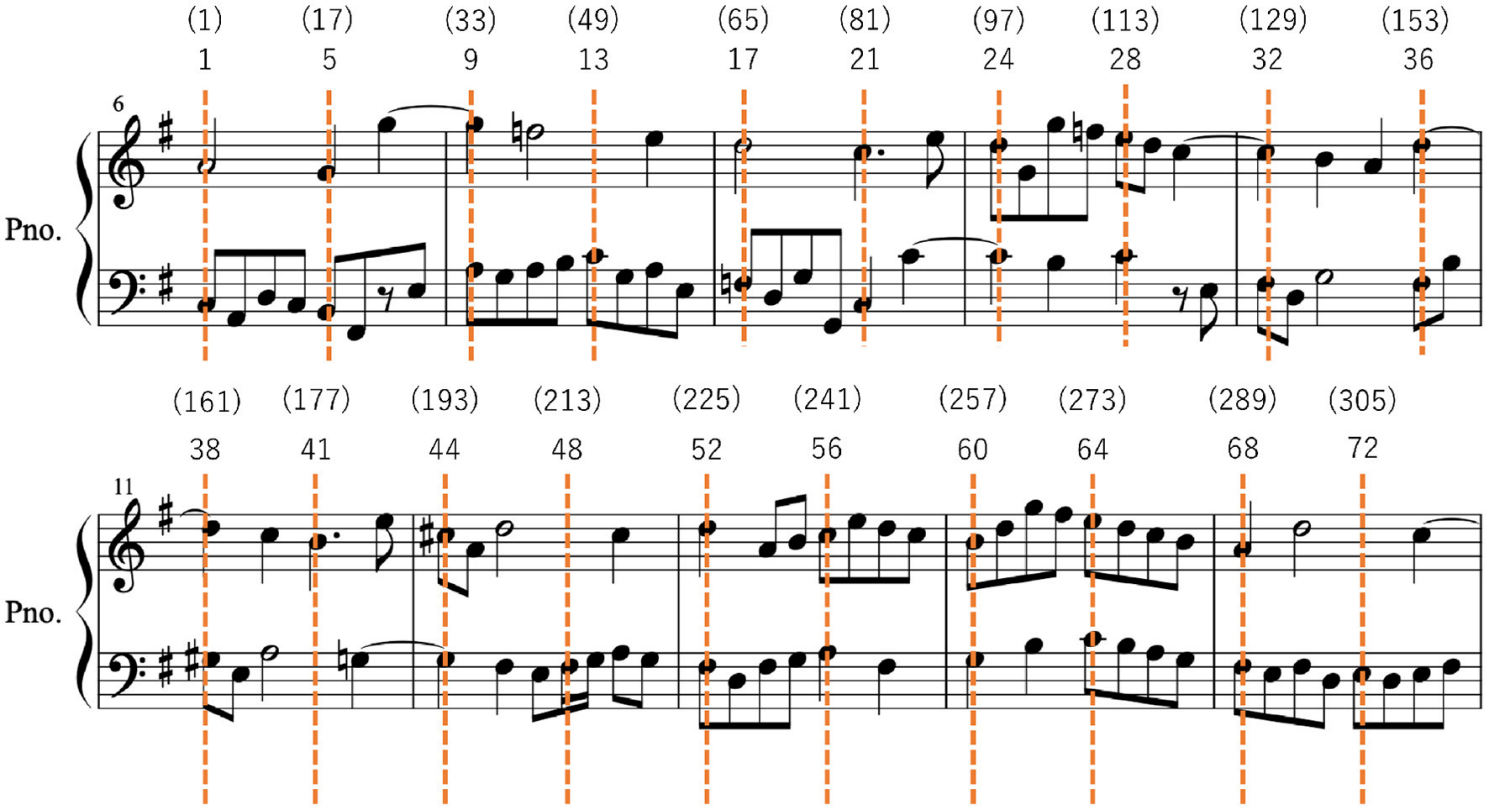
An example of numbering notes in the targeted portion (6–15 measures) for N-EHS (without parentheses) and B-EHS (with parentheses), using score LM. If the participant was playing G_4_ (in the treble clef) and B_2_ (in the bass clef) in measure 6 when her fixation on G_3_ (in the bass clef) in measure 7 ended, then N-EHS would be 5 (i.e., 10 – 5) and B-EHS would be 20 [ i.e., (37) – (17)].

The efficiency of visuo-motor coordination was measured by note-index eye-hand span (N-EHS) and beat-index eye-hand span (B-EHS), as well as the duration of sight-reading performance. For N-EHS, we first numbered each note as its numeric ID (e.g., 1, 2, 3) in the targeted portion (6–15 measures) of each score. Second, we identified the beginning and the ending time of each fixation. Finally, we identified a N-EHS by subtracting the numeric ID of a note being played at the time of the target fixation ended from the numeric ID of the target fixation (see Figure 2). For B-EHS, we numbered each beat, based on the thirty-second note (i.e., the shortest note used in the scores used) as the unit, as its numeric ID in the targeted portion of each score. The maximum of 32 beats per measure (4/4) and maximum of 320 beats at the end of the 15th measure. Finally, we identified a B-EHS by subtracting the numeric ID of a beat being played at the time of the target fixation ended from the numeric ID of the target fixation (see Figure 2). If the participant's fixation was located somewhere between two notes, its numeric ID was 0.5 point added to that of the left side. When the eye-tracking mark disappeared from the score (presumably because of the participant's checking of key or hand locations), we skipped measuring EHS until the eye-tracking mark reappeared on the score. For the duration of sight-reading performance (in ms), we calculated the duration between the first note of measure 6 being played and the last note of measure 15 being played.

#### Errors

The accuracy of execution was measured reversely by the number of errors. The errors were counted in each voice separately and added them together per score. Four types of errors were calculated in this study: the number of pitch errors (i.e., including missed, miss-played, or simultaneously played notes), the number of lengthened rhythm errors (i.e., notes played longer than what it should be), the number of shortened rhythm errors (i.e., notes played shorter than what it should be), and the number of stuttering (i.e., notes repeated unnecessarily).

#### Statistical analysis

Because most of data were not distributed normally, perhaps due to varying sight-reading skills among individuals as often indicated elsewhere (Sloboda, 1977; Lehmann and Ericsson, 1993; Underwood and Everatt, 1994; Meinz and Hambrick, 2010; Herrero and Carriedo, 2019), we used bootstrap *t*-tests with 1,000 iterations for all comparisons. To maintain the power of analysis, we set the level of significance by applying Bonferroni's correction for each comparison with the overall α for comparison to be 0.10 due to an exploratory nature of the study. In addition, Spearman's correlation coefficients were used to examine relations between dependent variables. All statistics were executed by SPSS (version 22).

## Results

Two pianists were invited to evaluate the performance level of the 32 participants, and a *t*-tests confirmed that there was no statistical difference in performance level between participants in the major (*n* = 16) and minor (*n* = 16) groups (*ps* > 0.10, *ds* = 0.24–0.50). Subsequently, we conducted a reliability analysis of the two pianists' evaluations, and the intraclass correlation coefficient confirmed that the evaluations showed reliability (*ICC* = 0.76).

### Effect of modality

We conducted bootstrap *t*-tests between major and minor conditions for four levels of intervallic complexity (i.e., low, medium, high, overall, subset α = 0.025 with Bonferroni's correction). We describe results according to three sight-reading phases.

#### Input

Table 2 shows means and standard deviations of three variables for an efficiency of input. The number of frames per fixation was equivalent (*ts*_*obt*_ (30) = −0.29 to 1.13, *ps* > 0.10, *ds* = 0.10–0.40) between major (*M* = 16.57, *SD* = 6.20) and minor (*M* = 14.84, *SD* = 5.69) conditions. However, the number of fixations was greater in minor (*M* = 126.04, *SD* = 54.19) than major (*M* = 63.38, *SD* = 25.80) condition, *t*_*obt*_ (30) = −4.18, *p* = 0.003, *d* = 1.48). Specifically, participants fixated significantly more in minor than major condition when the intervallic complexity was medium (*t*_*obt*_ (30) = −2.58, *p* = 0.009, *d* = 0.91) and low (*t*_*obt*_ (30) = −4.77, *p* = 0.002, *d* = 1.69). Even for a score with high intervallic complexity, this tendency appeared to be evident due to its medium effect size (*d* = 0.52). The proportion of fixation tended to be greater in major (*M* = 0.85, *SD* = 0.06) than minor condition (*M* = 0.76, *SD* = 0.10), *t*_*obt*_ (30) = 2.81, *p* = 0.02, *d* = 1.00). Specifically, participants fixated proportionately more in major than minor condition when the intervallic complexity was high (*t*_*obt*_ (30) = 3.02, *p* = 0.009, *d* = 1.07). Although statistically insignificant, medium to high effect sizes of modality on the proportion of fixation appeared to show the same tendency for medium (*d* = 0.66) and low (*d* = 0.89) intervallic complexities as well. An effect of modality was not observed in the duration of fixation.

**Table 2:** Means and standard deviations of three dependent variables for an efficiency of input, as well as results of bootstrap *t*-tests with Bonferroni's correction (*subset* α = 0.025) for effects of modality on each measure.

**Number of fixations**							
**Intervallic complexity**	**Major**		**Minor**		***t(30)***	***p***	***d***
	***M***	***SD***	***M***	***SD***			
High	72.81	29.78	88.56	31.00	−1.47	0.158^+^	0.52
Medium	60.19	30.47	88.88	32.45	−2.58	0.009**	0.91
Low	65.13	25.00	135.94	53.81	−4.77	0.002**	1.69
Overall	63.38	25.80	126.04	54.19	−4.18	0.003**	1.48
**Duration of fixation per frame**							
**Intervallic complexity**	**Major**		**Minor**		***t(30)***	***p***	***d***
	***M***	***SD***	***M***	***SD***			
High	14.60	5.48	15.24	7.09	−0.29	0.781	0.10
Medium	19.68	9.43	15.83	9.83	1.13	0.271	0.40
Low	17.18	7.70	14.66	4.72	1.12	0.327	0.40
Overall	16.57	6.20	14.84	5.69	0.82	0.437	0.29
**Proportion of fixation**							
**Intervallic complexity**	**Major**		**Minor**		***t(30)***	***p***	***d***
	***M***	***SD***	***M***	***SD***			
High	0.84	0.06	0.74	0.12	3.02	0.009**	1.07
Medium	0.84	0.08	0.76	0.16	1.85	0.078^+^	0.66
Low	0.88	0.14	0.77	0.12	2.52	0.051^+^	0.89
Overall	0.85	0.06	0.76	0.11	2.81	0.015*	1.00

**p < 0.01, *p < 0.025, ^+^p > 0.025 with medium to high effect size.

#### Visuo-motor coordination

Table 3 shows means and standard deviations of three variables for an efficiency of visuo-motor coordination. N-EHS was equivalent (*ts*_*obt*_ (30) = 0.44–1.27, *ps* > 0.10, *ds* = 0.16–0.45) between major (*M* = 3.12, *SD* = 1.33) and minor (*M* = 2.86, *SD* = 1.89) conditions. However, B-EHS indicated a medium effect size (*d* = 0.66) of modality with major (*M* = 13.75, *SD* = 5.81) appearing to be greater than minor (*M* = 9.74, *SD* = 6.30) condition. This tendency was evident in score of low intervallic complexity: B-EHS was greater in major than minor condition, *t*_*obt*_ (30) = 2.61, *p* = 0.018, *d* = 0.92). In addition, the duration of performance revealed less efficiency in minor condition. Participants spent significantly more time in sight-reading minor (*M* = 64.94, *SD* = 24.37) than major (*M* = 40.05, *SD* = 18.09) scores, *t*_*obt*_ (30) = −3.28, *p* = 0.003, *d* = 1.16). This difference was evident when the intervallic complexity was high (*t*_*obt*_ (30) = −2.32, *p* = 0.021, *d* = 0.82) and low (*t*_*obt*_ (30) = −4.32, *p* = 0.001, *d* = 1.53). Even sight-reading a score of medium intervallic complexity, this tendency was approaching significant with high effect size (*t*_*obt*_ (30) = −2.26, *p* = 0.026, *d* = 0.80).

**Table 3:** Means and standard deviations of three dependent variables for an efficiency of visuo-motor coordination, as well as results of bootstrap *t*-tests with Bonferroni's correction (*subset* α = 0.025) for effects of modality on each measure.

**N-EHS**							
**Intervallic complexity**	**Major**		**Minor**		***t(30)***	***p***	***d***
	***M***	***SD***	***M***	***SD***			
High	2.78	1.27	2.18	1.35	1.27	0.205	0.45
Medium	3.35	1.74	2.61	1.78	1.20	0.268	0.42
Low	3.37	1.43	2.89	1.85	0.81	0.409	0.29
Overall	3.12	1.33	2.86	1.89	0.44	0.654	0.16
**B-EHS**							
**Intervallic complexity**	**Major**		**Minor**		***t(30)***	***p***	***d***
	***M***	***SD***	***M***	***SD***			
High	12.95	6.64	10.29	7.02	1.10	0.277	0.39
Medium	13.60	7.61	11.15	8.31	0.87	0.403	0.31
Low	13.68	6.79	7.93	5.63	2.61	0.018*	0.92
Overall	13.75	5.81	9.74	6.30	1.87	0.067^+^	0.66
**Duration of performance**							
**Intervallic complexity**	**Major**		**Minor**		***t(30)***	***p***	***d***
	***M***	***SD***	***M***	***SD***			
High	39.56	18.98	55.46	19.85	−2.32	0.021*	0.82
Medium	41.15	18.67	55.78	17.94	−2.26	0.026^+^	0.80
Low	39.43	17.25	83.60	37.07	−4.32	0.001**	1.53
Overall	40.05	18.09	64.94	24.37	−3.28	0.003**	1.16

**p < 0.01, *p < 0.025, ^+^p > 0.025 with medium to high effect size.

#### Execution

Table 4 shows means and standard deviations of four variables for an accuracy of execution. No significant differences were found in any errors between major and minor conditions. However, medium to high effect sizes of modality were evident in three types of errors. More specifically, pitch errors appeared to be observed more in major than minor scores with high (*t*_*obt*_ (30) = 1.60, *p* = 0.207, *d* = 0.57) and medium (*t*_*obt*_ (30) = 1.67, *p* = 0.186, *d* = 0.59) intervallic complexities. In contrast, lengthened rhythm error and stuttering appeared to be observed more in minor than major scores, as evident in a medium effect size (*d* = 0.57) of modality for high intervallic complexity, and its high effect size (*d* = 0.87) for low intervallic complexity, respectively.

**Table 4:** Means and standard deviations of four dependent variables for an accuracy of execution, as well as results of bootstrap *t*-tests with Bonferroni's correction (*subset* α = 0.025) for effects of modality on each measure.

**Pitch error**							
**Intervallic complexity**	**Major**		**Minor**		***t(30**)*	***p***	***d***
	***M***	***SD***	***M***	***SD***			
High	10.88	18.90	3.25	2.35	1.60	0.207^+^	0.57
Medium	9.63	12.39	4.31	2.94	1.67	0.186^+^	0.59
Low	10.38	12.83	14.00	11.87	−0.83	0.420	0.29
Total	10.29	14.07	7.19	4.58	0.84	0.447	0.30
**Lengthened rhythm error**							
**Intervallic complexity**	**Major**		**Minor**		***t(30)***	***p***	***d***
	***M***	***SD***	***M***	***SD***			
High	0.94	1.29	1.88	1.93	−1.62	0.116^+^	0.57
Medium	2.19	2.34	1.81	2.20	0.47	0.644	0.17
Low	1.63	1.86	3.06	4.81	−1.12	0.274	0.39
Total	1.58	1.55	2.25	2.68	−0.86	0.396	0.30
**Shortened rhythm error**							
**Intervallic complexity**	**Major**		**Minor**		***t(30)***	***p***	***d***
	***M***	***SD***	***M***	***SD***			
High	1.25	2.21	1.50	1.26	−0.39	0.697	0.14
Medium	1.50	2.94	1.38	1.36	0.15	0.878	0.06
Low	1.13	1.59	1.94	3.23	−0.90	0.374	0.32
Total	1.29	2.16	1.60	1.35	−0.49	0.624	0.17
**Stuttering**							
**Intervallic complexity**	**Major**		**Minor**		***t(30)***	***p***	***d***
	***M***	***SD***	***M***	***SD***			
High	4.88	7.54	6.00	6.39	−0.46	0.652	0.16
Medium	5.94	7.59	4.94	7.25	0.38	0.706	0.14
Low	4.38	4.53	11.81	11.19	−2.46	0.036^+^	0.87
Total	5.06	6.05	7.58	7.72	−1.03	0.327	0.36

**p < 0.01, *p < 0.025, ^+^p > 0.025 with medium to high effect size.

### Relations among three phases of sight-reading process

Table 5 shows Spearman's correlation coefficients between all dependent variables. We summarize the results first relations between variables of each phase, and then move on to those between phases.

**Table 5:** The Spearman's correlation coefficient among variables of Input, Visuo-motor coordination, and Execution.

		**Input**			**Visuo-motor coordination**			**Execution**			
			**DF**	**PF**	**N-EHS**	**B-EHS**	**DP**	**PE**	**LRE**	**SRE**	**St**
	Input	NF	−0.45**	−0.66**	−0.33	−0.49**	0.80**	0.19	0.25	−0.01	0.42*
			DF	0.55**	−0.07	0.07	0.003	−0.36*	0.14	0.01	0.10
				PF	−0.02	0.15	−0.55**	−0.15	−0.02	−0.05	−0.10
				Visuo-motor coordination	N-EHS	0.91**	−0.39*	−0.03	0.06	0.29	−0.07
						B-EHS	−0.50**	−0.11	−0.03	−0.20	−0.19
							DP	0.14	0.38*	0.10	0.53**
							Execution	PE	0.53**	0.39*	0.34
									LRE	0.43*	0.33
										SRE	0.67**

*p < 0.05, **p < 0.01.

*NF, Number of fixations; DF, Duration of fixation per frame; PF, Proportion of fixation; N-EHS, Note-index eye-hand span; B-EHS, Beat-index eye-hand span; DP, Duration of performance; PE, Pitch error; LRE, Lengthened rhythm error; SRE, Shortened rhythm error; St, Stuttering*.

#### Input

The number of fixations (NF) was correlated negatively with the duration of fixation (DF, *r*_*s*_ = −0.45, *p* = 0.010) and the proportion of fixation (PF, *r*_*s*_ = −0.66, *p* < 0.001). The proportion of fixation (PF) and the duration of fixation (DF) were positively correlated (*r*_*s*_ = 0.55, *p* = 0.001). This indicates that the more the duration of fixation, the fewer number of fixations, and the greater the proportion of fixation.

#### Visuo-motor coordination

Two indices of EHS were correlated positively (*r*_*s*_ = 0.91, *p* < 0.001), and this high correlation demonstrates that both N-EHS and B-EHS measure the same construct. The duration of performance (DP) was correlated negatively with N-EHS (*r*_*s*_ = −0.39, *p* = 0.029) and B-EHS (*r*_*s*_ = −0.50, *p* = 0.004). These results suggest that the longer the EHS, the shorter the duration of sight-reading performance.

#### Execution

The analyses confirmed moderate to high correlations between the different types of performance errors. Among them, pitch errors (PE) were correlated positively with both shortened (SRE, *r*_*s*_ = 0.39, *p* = 0.025) and lengthened (LRE, *r*_*s*_ = 0.53, *p* = 0.002) rhythm errors, whereas stuttering (St) was correlated positively only with shortened rhythm error (*r*_*s*_ = 0.67, *p* < 0.001). Only moderate positive correlation between two types of rhythm errors (*r*_*s*_ = 0.43, *p* = 0.014) indicates that these measures can tap into different aspects of performance errors, which may not be revealed otherwise.

#### Input vs. visuo-motor coordination

Of those, the duration of performance—representing an inefficiency of visuo-motor coordination—was correlated positively with the number of fixations (*r*_*s*_ = 0.80, *p* < 0.001) and negatively with the proportion of fixation (*r*_*s*_ = −0.55, *p* = 0.001). B-EHS (*r*_*s*_ = −0.49, *p* = 0.004)—representing an efficiency of visuo-motor coordination—was correlated negatively with the number of fixations. These results indicate that inefficiencies during input (i.e., more frequent, or less proportion of, fixations) are related to a slower tempo and shorter EHS during visuo-motor coordination of music sight-reading.

#### Input vs. Execution

The number of fixations was correlated positively with stuttering (*r*_*s*_ = 0.42, *p* = 0.016) and the duration of fixation was correlated negatively with pitch error (*r*_*s*_ = −0.36, *p* = 0.040). This indicates that an inefficient input (i.e., more frequent fixations or shorter duration of fixation) is related to a poor execution (i.e., more stuttering or pitch error) in music sight-reading. When the input processing becomes inefficient, the duration of each fixation gets shorter, and less note information can be acquired. To ensure uninterrupted playing, the performance is kept even if there are successive wrong notes, resulting in more pitch errors.

#### Visuo-motor coordination vs. execution

The duration of performance (DP) was correlated positively with shortened rhythm error (SRE, *r*_*s*_ = 0.38, *p* = 0.030) and stuttering (St, *r*_*s*_ = 0.53, *p* = 0.002) while N-EHS and B-EHS was not correlated with any errors. This indicates that an inefficient visuo-motor coordination measured by the duration of performance (i.e., a slower performance) is related to a shortened rhythm error or stuttering during execution of music sight-reading.

## Discussion

The primary goal of this study was to investigate effects of modality in three phases of music sight-reading: input, visuo-motor coordination, and execution. From the literature, skilled sight-reading requires both *accuracy* (Waters et al., 1997; Gudmundsdottir, 2010; Adachi et al., 2012; Herrero and Carriedo, 2019) and *efficiency* (Sloboda, 1974; Goolsby, 1994; Truitt et al., 1997; Madell and Hébert, 2008; Cara, 2018), and these key concepts need to be considered in discussing what is going on during each phase of sight-reading process. In reality, however, the accuracy is observable only during execution even though we are aware that inaccurate input or missed visual-to-motor conversion is also possible. The present study is not free from this dilemma; we will primarily discuss efficiency regarding input and visuo-motor coordination while discussing accuracy regarding execution. At least, correlational analyses between variables across phases allow us to discuss relations between accuracy and efficiency.

### Modality and intervallic complexity in accuracy and efficiency of music sight-reading

Overall, modality influenced input in the way we predicted. During input, sight-readers fixated more frequently (i.e., encoded information more inefficiently) for minor than major scores. The greater proportion of fixation for major than minor scores means that sight-readers spent more time looking at notes and less time looking down at the keyboard for major scores. Even though this tendency was apparent in sight-reading a score with the high intervallic complexity, similar tendencies were observed also for other scores, which appears to imply that features unique to a minor score (e.g., embedded accidentals) may be more responsible for inefficiency than its intervallic complexity.

During visuo-motor coordination, on the other hand, a significant interaction between modality and intervallic complexity was observed: B-EHS was greater in sight-reading a major than minor score only with low intervallic complexity. This appears to have derived from much smaller B-EHS in sight-reading a minor score with low intervallic complexity (i.e., 7.93) relative to the rest of scores (i.e., 10.29–13.68, see Table 3). This contradicts our prediction that a higher intervallic complexity would result in lower efficiency during visuo-motor coordination. In addition, a slower performance (i.e., inefficient visual-to-motor conversion of information) was evident in sight-reading minor than major scores regardless of their intervallic complexities, again, contradicting our prediction. How did this happen?

One obvious explanation is that we did not control *tempo* (or speed) of performance. Slowing down for minor scores would have helped sight-readers maintain their efficiencies for a more difficult task (Truitt et al., 1997; Furneaux and Land, 1999; Wurtz et al., 2009; Lewandowska and Schmuckler, 2020), reflected in equivalent sizes of N-EHS among all scores (i.e., 2.18–3.37, see Table 3). Based on the collected analyses of the sight-reader's assessment of the score, we found that modality did not appear to influence the sight-reader's assessment of the score (*t*_*obt*_ (30) = −1.79, *p* = 0.088, *d* = 0.63). That is, the sight-reader seemed to perceive no difficulty in sight-reading scores in either major (*M* = 4.46, *SD* = 1.45) or minor (*M* = 5.42, *SD* = 1.58). Such results confirm the above explanation that when the efficiency is reduced, the sight-reader performs relatively difficult tasks more easily by lowering their tempo. This rationale can also explain equivalent sizes of B-EHS between scores of medium and high intervallic complexities, but we still need an alternative explanation for the smaller B-EHS of minor score with low intervallic complexity.

In the present study, we controlled various factors of sight-reading materials such as intervallic complexities, meter, and the total number of measures, but we overlooked density of notes within a measure. The minor score with low intervallic complexity consisted of many more 16th notes than its major counterpart. This unbalanced density may have served as a confounding variable for the paired scores of low intervallic complexities. The higher note density (or many more 16th notes) of the minor score with low intervallic complexity can also explain its extremely greater number of fixations (i.e., 135.94) relative to those for other scores (i.e., 60.19–88.88) during input, and that of stuttering (i.e., 11.81) relative to other scores (i.e., 4.38–6.00) during execution.

Furthermore, as predicted, the modality did not have any effect on the accuracy and flow of the execution. There were no significant differences between major and minor scores for any performance error, although the modality influenced the input. This implies that, even though the sight-reader's input was less efficient in identifying the minor score, they improved psychological stability and reduced pitch errors by slowing down the tempo without being controlled (Lewandowska and Schmuckler, 2020).

It is noteworthy that those sight-readers who played faster seemed to make more pitch errors. Why does this occur? One plausible explanation is that the faster tempo leaves the sight-reader with no more response time and is more likely to make pitch errors. In order to keep the performing intact, sight-reader does not make error corrections, and usually deviations in one note lead to pitch errors in several consecutive notes (and possibly several phrases or measures) that follow. This seems to indicate that a sight-reader who plays fast is not necessarily an accurate sight-reader (Cara, 2018).

However, the complexity of interval did not seem to affect the accuracy and flow during the sight-reading as we predicted, only showing some trends. We observed a tendency to show fewer pitch errors when sight-reading minor scores with high and medium intervallic complexity (i.e., 3.25–4.31, see Table 4) than major (i.e., 9.63–10.88, see Table 4). In high intervallic complexity, major (i.e., 0.94, see Table 4) showed a tendency to make fewer lengthened rhythm errors than minor (i.e., 1.88, see Table 4). As previously mentioned, these trends seem to imply that tempo may be more influential on performance accuracy than the complexity of the interval.

### Correlation of efficiency with accuracy and flow of music sight-reading

In general, the results of the analysis were in line with our predictions. That means, the shorter the performing duration, the less the lengthened rhythm error and stuttering. This suggests that efficient visuo-motor coordination ensure accuracy and flow of execution. It should be noted that although we obtained a positive correlation (i.e., *r*_*s*_ = 0.38–0.53), this does not contradict our prediction (i.e., efficiency is negatively correlated with accuracy and flow). This is because when the visuo-motor coordination is inefficient, the sight-reader cannot mobilize the fingers to play the corresponding keys accurately, they try to obtain the latency time by reducing the tempo. This leads to longer duration of performance and less accuracy and flow.

Unfortunately, however, although our predictions were confirmed, we did not see a correlation between accuracy and flow of execution and EHS. However, the significant negative correlation between EHS and performance duration indicated that the longer the duration of the performance, the shorter the EHS. We can explain this by the fact that the sight-reader expects to look forward to getting more musical information, but the present study did not control the tempo for as close to natural sight-reading as possible, and the sight-reader could perform at a tempo that suited them. That means, when visual-motor coordination is inefficient, sight readers are unable to successfully motivate finger movements through visual information and can only adjust to their sight-reading by slowing down the tempo, which led us to find no correlation between performance errors and EHS. This seems to indicate that when visual-motor coordination processing is inefficient, the sight-reader tries to balance the efficiency of information conversion and accuracy of execution by slowing down the tempo and narrowing the EHS (Rosemann et al., 2016).

On the other hand, the significant correlation between input and visuo-motor coordination showed that by spending more time looking at the notes (i.e., an increased proportion of fixation) and using less fixation, the sight-reader increased the efficiency of musical information input, increased the B-EHS, converted visual information into finger movement in a timely manner, enabling perform fluency even with faster tempo, reduced performance duration, and improved visuo-motor coordination efficiency. Sight-reading is primarily determined by effective visual input and the process of visual-to-motor conversion of musical notation information (Waters et al., 1998). For advanced pianists, an increase of fixations is accompanied by an increase of performing time (Chang, 1993). This suggests that inefficient input leads to a failure of visuo-motor coordination. Because the sight-reader cannot input musical information efficiently enough to convert visual information into finger movements smoothly but must slow down the tempo. Sight-reading requires playing the right rhythmic structure at the right time (Drake and Palmer, 2000). The correlation between the variables of input processing supports that when there are no rhythmic constraints, sight-readers can play at a tempo that suits them, without having to rush to expect musical information too far ahead of the playing position. The low load during information input allows the sight-reader to reduce unnecessary eye movements by holding fixations for long periods of time to ensure that more musical information is available with each fixation, such as by reducing the number of fixations that must be paused to gaze at the score in order to check the keyboard position with the head down (as reflected in the increased proportion of fixation).

Moreover, the significant correlation between input and execution showed that the more fixations and the shorter the duration of per fixation, the more likely it was to produce pitch errors and stuttering. This suggests that more frequent eye movements lead to inefficient input, as well as low accuracy and stuttering of execution.

In addition, we noticed a significant positive correlation between accuracy (i.e., error) and fluency (i.e., stuttering) of the performance. This may be due to the sight-reader's perception that the score is a little difficult or a lack of confidence in the performance (Adachi et al., 2012). This suggests that when sight-readers realize that a performance error has occurred, they tend to correct it repeatedly starting from the neighboring note where the performance error occurred. Sometimes, in order to achieve a more perfect sight-reading, sight-reader will also correct from the first note of the measure where the performance error occurred until they are satisfied (McPherson, 1994). Furthermore, we found that sight-players were more likely to make pitch errors rather than rhythm errors. This may be because the sight-reader tries to play the notes at the correct time, rather than playing each note correctly. Sight-players consider that playing notes fluently is more important than playing notes accurately from the score (Drake and Palmer, 2000; Lehmann and McArthur, 2002).

## Conclusion

This article evaluated the modality of music material (major mode and minor mode) would affect the input processing, the visuo-motor coordination processing, and the execution during sight-reading, as well as the effect of interval complexity on the three phases of sight-reading. In addition, the correlation among these three phases of processes is explored. Thirty-two advanced pianists sight-read three two-voice Baroque pieces in either major mode or minor mode and recorded their eye movements by an eye tracker. The results showed that modality of music material have an impact on the efficiency of input processing and visuo-motor coordination processing in the sight-reading. Sight-readers used more fixation in minor scores than in major scores and tended to sight-read minor scores longer. Moreover, the higher the interval complexity, the less efficient the information processing during sight-reading. In addition, because it is demonstrated that there is a correlation among these three phases of information processes, indicated that an efficient input processing and an efficient visuo-motor coordination processing would represent an accurate execution in sight-reading.

The current study makes the relation between the series of information processing in sight-reading more clearly and confirmed that the modality of music could affect the efficiency of information processing during sight-reading. There was no other study has considered in the experiment before. It should be noted that this study was implemented under the premise of uncontrolled tempo, thus the sight-reader could play with their own tempo. If the tempo is under controlled, how the modality of score affects these three phases of information processing in sight-reading should be explored in future research. In addition, this study only used Baroque music materials as the stimulus, however, whether the modality influence other types of music (e.g., the Classical music; the Romantic music, the 20th century music) has not studied yet and undoubtedly requires further research.

## Data Availability Statement

The original contributions presented in the study are included in the article/supplementary material, further inquiries can be directed to the corresponding author/s.

## Ethics Statement

The studies involving human participants were reviewed and approved by Hokkaido University Institutional Review Board. The patients/participants provided their written informed consent to participate in this study.

## Author contributions

JQ and MA contributed to conception and design of the study. JQ performed experiment, organized the database, performed the statistical analysis, and wrote the first draft of the manuscript. MA wrote sections of the manuscript. All authors contributed to manuscript revision, read, and approved the submitted version.

## Funding

This work was supported by the Music Psychology Laboratory of the Graduate School of Humanities and Human Sciences, Hokkaido University, Japan.

## Conflict of interest

The authors declare that the research was conducted in the absence of any commercial or financial relationships that could be construed as a potential conflict of interest.

## Publisher's note

All claims expressed in this article are solely those of the authors and do not necessarily represent those of their affiliated organizations, or those of the publisher, the editors and the reviewers. Any product that may be evaluated in this article, or claim that may be made by its manufacturer, is not guaranteed or endorsed by the publisher.
